# The Effects of Cooking Methods on Gel Properties, Lipid Quality, and Flavor of Surimi Gels Fortified with Antarctic Krill (*Euphausia superba*) Oil as High Internal Phase Emulsions

**DOI:** 10.3390/foods13244070

**Published:** 2024-12-17

**Authors:** Yinyin Lv, Xiuqin Wang, Ruoyi Hao, Xianhao Zhang, Xianbing Xu, Shengjie Li, Xiuping Dong, Jinfeng Pan

**Affiliations:** 1 State Key Laboratory of Marine Food Processing and Safety Control, National Engineering Research Center for Seafood, Collaborative Innovation Center of Provincial and Ministerial Co-Construction for Seafood Deep Processing, Liaoning Province Collaborative Innovation Center for Marine Food Deep Processing, Dalian Technology Innovation Center for Chinese Pre-made Food, College of Food Science and Technology, Dalian Polytechnic University, Dalian 116034, China; 13931053789@163.com (Y.L.); wxq223@163.com (X.W.); zhangxianhao98@hotmail.com (X.Z.); xianbingxu@gmail.com (X.X.); shengjie.li2016@outlook.com (S.L.); dxiuping@163.com (X.D.); 2 Department of Food Science and Technology, School of Forestry, Beihua University, Jilin 132013, China; ruoyi_hao@163.com

**Keywords:** Antarctic krill oil, emulsion gel, cooking method, gelling properties, flavor attributes, fish protein

## Abstract

In this study, silver carp surimi products enriched with Antarctic krill oil high internal phase emulsions (AKO-HIPEs) were cooked using steaming (STE), microwave heating (MIC), and air-frying (AIR), respectively. The gel and flavor properties, lipid quality and stability were investigated. Compared to the MIC and AIR groups, the STE surimi gel added with HIPEs had better texture properties, exhibiting higher water-holding capacity and a more homogeneous structure, while the air-frying treatment resulted in visually brighter surimi products. The degree of lipid oxidation during cooking was in an order of STE < MIC < AIR as determined by electron paramagnetic resonance spectrometer and thiobarbituric acid reactive substances. HIPE-added surimi gels retained more nutrients and flavor when cooked by AIR compared to STE and MIC. Results imply that the texture properties and lipid stability of surimi products fortified with AKO-HIPEs were better than those of the oil group under any cooking method. In conclusion, surimi products added with AKO-HIPEs had better gel properties and retained more fatty acids and flavor than AKO-SO.

## 1. Introduction

With a large proportion of protein and resilient properties, surimi products are popular due to their high nutritional value and special texture attribute [1]. In the post-pandemic era, food nutrients and their effects on human health are attracting unprecedented attention. Polyunsaturated fatty acids are closely related to human health, particularly n-3 polyunsaturated fatty acids (n-3 PUFAs), which are extremely important for infant brain development and cognitive ability development [2]. Meanwhile, a large number of studies have confirmed that n-3 PUFAs play positive roles in preventing and alleviating cardiovascular and cerebrovascular diseases [3], cancer [4], and inflammation [5]. Therefore, the addition of oils containing large proportion of n-3 PUFAs such as fish oil [6], flaxseed oil, and seaweed oil [7] into surimi products is appealing. It has been noticed that the method of oil addition is vital to the physical properties, sensory quality, and nutritional value of final surimi products.

High internal phase emulsions (HIPEs) are emulsions with a volume enrichment of the internal dispersed phase higher than 74% [8]. HIPEs are able to transfer liquid oils and fats into a semi-solid gel structure and have a high efficiency of encapsulation of the active substance [9,10]. Studies showed that HIPEs are less susceptible to lipid oxidation mainly due to their high accumulation oil phase, which isolates oxygen [11]. And the interfacial film produced by emulsification acts as a physical or electrostatic barrier to prevent droplets collapse and oxygen entry into the oil phase [12]. Therefore, HIPEs are excellent candidates for enhancing lipid in surimi products for their high stability and lipid-loading capacity as well as low oxidation susceptibility. They might mitigate the negative impact of lipid incorporation on myofibrillar protein gel network.

As an essential part during the processing of surimi products, cooking is not only crucial for safety but also for the quality (color, flavor, texture, etc.) of final products. Two-step heating is the commonest method for surimi cooking, which reduces protein degradation by endogenous proteases and improves the gel properties of surimi product. Microwave heating is the conversion of electromagnetic energy into thermal energy through the interaction of material components with electromagnetic waves [13], which is characterized by green processing, fast and targeted heating, and high efficiency [14]. Containing high proportion of moisture and salt, surimi has a strong dielectric response and microwave absorption due to dipole polarization and ionic conduction [15], suitable for heating by microwave. Research demonstrated that microwave-heated surimi exhibited increased myosin cross-linking, which in turn led to the formation of a dense network structure and enhanced gel strength [16]. As a new type of food-frying technology, air-frying can evenly distribute the heat on food surface by spraying hot air around [17]. It is reported that air-frying reduces the generation of harmful substances, such as trans fatty acids, while being able to provide a similar appearance and color to conventional frying [18]. Further, the thorough contact of lipid with air in air-frying could promote lipid oxidation to produce more flavor compounds (aldehydes and ketones) [17].

Our previous study showed that Antarctic krill (*Euphausia superba*) oil high internal phase emulsions (AKO-HIPEs) could fortify n-3 PUFAs to enhance the nutritional value of surimi products while maintaining fairly good gel properties [19]. Heating is a key process to induce the formation of surimi quality. Thus, this study aimed to expose the effects of different cooking methods, including steaming, microwave heating, and air-frying, on the physical properties, lipid quality, and flavor attributes of surimi gels incorporating AKO-HIPEs, so as to provide useful information toward surimi product innovation.

## 2. Materials and Methods

### 2.1. Materials

AAA-grade frozen silver carp surimi was purchased from Honghu Jingli Aquatic Products & Foodstuff Co., Ltd. (Wuhan, China). Antarctic krill oil containing ≥50 g/100 g phospholipids, ≥200 mg/kg astaxanthin, and ≥22% n-3 PUFA was purchased from Kangjing Marine Biotechnology (Weifang, China). Soybean oil (SO) was acquired from the local supermarket (Dalian, China). All the other chemicals were analytical grade.

### 2.2. Experimental Design and Preparation for AKO-HIPEs and Surimi Sols

Surimi gels fortified with lipid as Oil or HIPE were cooked using three heating methods. The experimental design is shown in Table 1.

AKO-HIPEs were prepared as in a previous method [20]. Briefly, casein solution (2%, pH = 6) and complex oil (30% AKO and 70% SO) were mixed with 75% oil phase volume ratios. The mixture was continuously homogenized with a high-speed digital homogenizer (Ultra-Turrax T10, IKA, Staufen, Germany) at 9000 rpm for 120 s at room temperature to form AKO-HIPEs. The AKO-HIPEs were kept at 4 °C until use.

Surimi sol was prepared according to a previous method [19]. Briefly, frozen surimi was thawed, cut, and chopped using a Midea MJ-MC05Q1-403 cutter (Foshan, China) at 8000 rpm/min for 2 min, involving four cycles of chopping and mixing for 30 s each. Ice water was introduced to adjust the surimi moisture to 80% (*w*/*w*). Subsequently, 2.5% NaCl was added into surimi paste and chopped for 2 min. Different forms of AKO (HIPE group: 7.34 g AKO-HIPEs/100 g surimi; oil group: 5.50 g AKO-SO/100 g surimi) were added to the surimi paste. The same proportion of casein and water as the quantity of AKO-HIPEs was added into the corresponding oil groups to achieve the same formula. The samples were chopped for another 2 min. The whole process was performed at a temperature below 10 °C. Thereafter, surimi sols were stuffed into polycarbonate flat-bottom tubes (diameter 20 mm) and sealed until further cooking.

### 2.3. Cooking of Surimi Gels

The surimi sols from Section 2.2 were incubated at 40 °C for 30 min and cooked using different cooking methods: steaming (vapor, 6 min) by an CM101 universal steam oven (Rational, Schmalzturm, Germany); microwave heating (340 W, 100 s) by a NE-1753 microwave oven (Panasonic, Osaka, Japan); air-frying (180 °C, 6 min) by a MF-KZ42E101 air fryer (Midea, Shunde, China). The cooking conditions of the three methods were decided by the sensory quality of the cooked samples after a series of optimization tests. The cooked gels were stored at 4 °C for tests.

### 2.4. Gel Properties Analyses

#### 2.4.1. Textural Properties

The textural properties of the gels were measured according to Zhang et al. [21] with modifications. The gels were analyzed using a TA.XT.plus texture analyzer (Stable Micro Systems, Surrey, UK) at 50% strain and 5 g of trigger force equipped with P/50 probe. Measurement was performed as follows: pre-test speed = 1 mm/s; test speed = 1 mm/s; return speed = 2 mm/s. Texture parameters, including hardness, chewiness, resilience, cohesiveness, and springiness, were calculated.

#### 2.4.2. Cooking Loss (CL)

After heating, the released liquid on surface of gels was wiped off with filter paper. *CL* was calculated as below:

CL=(G0−G1)/G0×100%

where *G*0 is the mass of surimi paste before cooking and *G*1 is the mass of the surimi gel after the liquid expelled has been wiped away.

#### 2.4.3. Color Properties

The color characteristics of gels were assessed using an UltraScan Pro spectrometer (HunterLab, Reston, USA). The L* value denoting lightness, a* denoting red (+) and green (−), and b* denoting yellow (+) and blue (−) were assessed.

#### 2.4.4. Water Distribution

Water distribution in gel was investigated by low-filed NMR (LF-NMR) according to Cen et al. [22] with adjustments. Approximately 10 g surimi gel was placed into NMR tubes. T2 relaxation time was obtained by Carr–Purcell–Meiboom–Gill (CPMG) sequence in a MesoQMR23-060H LF-NMR (Niumag Analytical Instrument Co., China). TW = 4500 ms; TE = 0.5 ms; PRG = 1. Data were analyzed using MultiExp Inv. Analysis software (v2.0, Niumag Analytical Instrument Co., Suzhou, China). The hydrogen proton density images of the samples were collected using magnetic resonance imaging (MRI) software (v2.0, Niumag Analytical Instrument Co., Suzhou, China). A multi-spin echo imaging sequence was used.

#### 2.4.5. Gel Microstructure

The microstructure was detected according to Yan et al. [23] with modification. The samples were cut into 2 mm thick slices and fixed in 4% formaldehyde fixative for 24 h, followed by dehydration and fixation and paraffin embedding. The samples were sectioned, dewaxed, and then stained with hematoxylin and eosin. The final sections were dehydrated and sealed. Microstructures of gels were imaged on an Upright optical microscope (Nikon Eclipse E100, Olympus Optical Co., Tokyo, Japan) mounted with a digital camera.

### 2.5. Lipids Properties Analyses

#### 2.5.1. Fatty Acid Composition

Lipids were extracted as in Hao et al. [24]. Briefly, lipids in surimi gel were extracted using a mixture of n-hexane and isopropyl alcohol in a 3:2 ratio (*v*/*v*) through two consecutive rounds of extraction. The lipids were methylated using the BF3–methanol method and FA profile analysis was conducted as in Wang et al. [25]. Samples were filtered through a 0.22 µm organic filter membrane and analyzed by gas chromatograph (Agilent 7890B, Foster City, CA, USA) equipped with a Supelco SP2560 capillary column (100 m × 0.25 mm, 0.2 µm). By comparing the retention times of the standards, the fatty acids in the samples could be identified and quantified.

#### 2.5.2. Astaxanthin Content

Astaxanthin was extracted and analyzed as in Lv et al. [19]. Briefly, surimi gels were homogenized with ethyl acetate to extract astaxanthin. The mixture was centrifuged, and supernatant was collected. The absorbance of the extracts at 474 nm was determined. Standard curves were prepared and astaxanthin content was calculated.

#### 2.5.3. Thiobarbituric Acid Reactive Substances (TBARSs)

The TBARS value was used to evaluate the lipid oxidation of surimi gel as in Pang et al. [26]. Surimi gels were homogenized with 5% trichloroacetic acid. After centrifuging the mixture, the supernatant was collected and prepared for testing. The samples were incubated with thiobarbituric acid and the absorbance at 532 nm was measured. Malondialdehyde (MDA) content was determined using a standard curve. The TBARS value is expressed as milligrams of malondialdehyde (MDA) per kilogram of surimi gel.

#### 2.5.4. Free Radicals

Free radicals in surimi gel were detected by an electron paramagnetic resonance spectrometer (ESR). Freeze-dried surimi gel powder was placed into a 5 mm glass capillary tube, and ESR spectra were measured by an ESR spectrometer (Bruker A200, Karisruhe, Germany). The test conditions were based on Qi et al. [27]. The signal intensity was determined by calculating the average value of the absolute values of the high and low peak signals. The experiment was triplicated.

### 2.6. Electronic Nose Analysis

The flavor pattern of samples was determined by a portable electronic nose system (PEN3, Win Muster Airsense Analytics Inc., Schwerin, Germany). Briefly, 3 g samples were placed into 20 mL vials and capped immediately. The samples were analyzed under the following conditions: measurement time = 80 s; flow rate = 300 mL/min; flushing time = 30 s. The data were collected and analyzed using the included software. The electronic nose results are presented as a radar diagram and PCA plots [28].

### 2.7. Sensory Evaluation

The sensory evaluation of surimi gel was conducted by quantitative descriptive analysis (QDA). The samples were placed in a white ceramic dish and were equilibrated at 25 °C for 20 min. The surimi gels were randomly encoded with three digits and randomized complete block designs (RCBDs) were used to lower or block the deviation among the panelists. The evaluators were twenty trained food students (age: 20 to 30) who were familiar with surimi products. After moderate discussion, five sensory properties and their corresponding scoring criteria were developed, as shown in Table 2. The evaluators scored the sensory properties of the surimi gel items by item following the sensory evaluation criteria. After completing one sample evaluation, each evaluator rinsed their mouth with purified water and rested for 1 min before evaluating the next sample. After the evaluation, the score sheet was collected, and the experimental results were counted.

### 2.8. Statistical Analysis

Experimental results were replicated at least three times. Data were expressed as mean ± standard deviation (STD) and submitted to one-way analysis of variance (ANOVA). Means were compared with Duncan’s multiple range tests using software SPSS 19.0 (SPSS Inc., Chicago, IL, USA). If *p* < 0.05, difference was defined as significant.

## 3. Results

### 3.1. Physical Changes

#### 3.1.1. TPA

**Figure 1:**
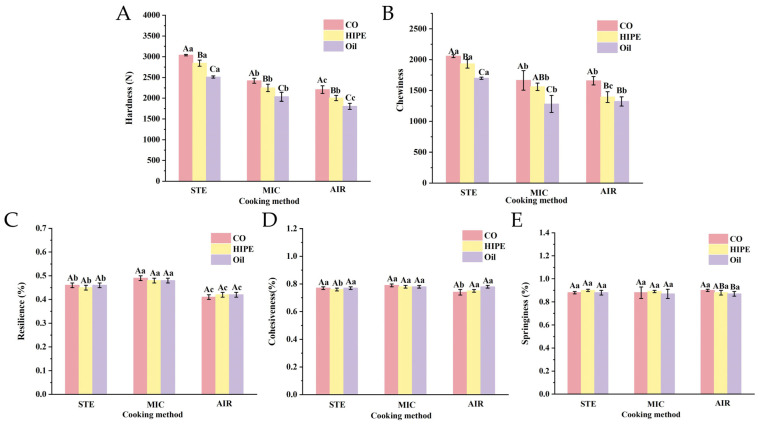
The hardness (**A**), chewiness (**B**), resilience (**C**), cohesiveness (**D**), and springiness (**E**) of surimi gels in different cooking methods. Uppercase letters denote statistical differences between groups of different addition methods in the same cooking method. Lowercase letters denote statistical differences between groups of different cooking methods in the same addition method (*p* < 0.05). CO: no AKO added to surimi gel; HIPE group: AKO-HIPEs were added to surimi gel; oil group: AKO-SO were added to surimi gel. STE: steamed; MIC: microwave heated; AIR: air-fried.

In Figure 1A,B, the hardness and chewiness of the MIC and AIR groups were significantly lower than those of the STE groups (>2400 g and 1699, respectively). These indicated that the surimi gels obtained by STE have better textural properties. The steaming heats the surimi gel by stable and even steam, maintaining it in a moist environment with a moisture equilibrium. This approach minimizes water loss and limits disruption to the gel structure, thus maintaining a better gel properties. Microwave heats from the inside of food, which would cause fast water dissipation from the gel, resulting in gel network disruption. Meanwhile, air-frying allows for hot air to circulate rapidly through the airtight oven, taking away the moisture on the surface of gel. During this period, water would rush out of the gel network, causing mechanical damage to the gel structure. The resilience of the MIC groups was higher than that of the STE and AIR groups (Figure 1C, *p* < 0.05). It may be that the microwave provides sufficient energy within a short time to propel the cross-linking of myofibrillar protein (MP) molecules, thereby forming a fairly good gel structure [29]. Since the MIC gel contained less moisture content, it showed slightly higher resilience. No significant differences in springiness and cohesiveness were observed in samples that used different cooking methods (Figure 1D,E).

As expected, the hardness and chewiness of the HIPE and oil group were lower than that of the control (CO) using the same cooking method. All HIPE groups exhibited higher hardness and chewiness than their oil counterparts (Figure 1A,B). It is known that oil addition blocks the cross-linking of MP due to the heavy aggregation of oil droplets, inhibiting the gelation process and giving a poor gel texture [1]. However, HIPE droplets are stable, and they could distribute in surimi evenly and mitigate the influence of oil on MP gelation. Additionally, HIPE droplets could fill into the voids in gel network to restrict the movement of the gel matrix more effectively than oil droplets [19]. These might help HIPE samples to form gels with strong networks and good textural properties.

#### 3.1.2. Cooking Loss

**Figure 2:**
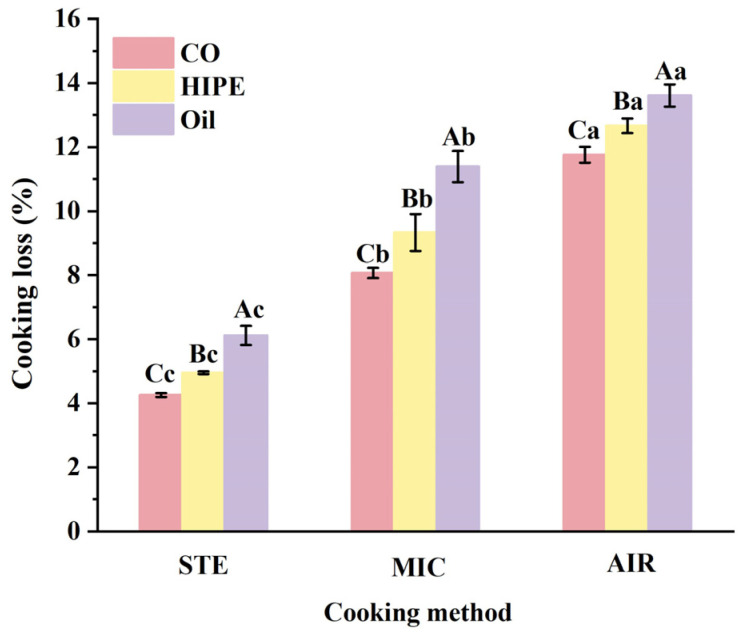
The cooking loss of surimi gels using different cooking methods. Uppercase letters denote statistical differences between groups of different addition methods in the same cooking method. Lowercase letters denote statistical differences between groups of different cooking methods in the same addition method (*p* < 0.05).

CL mainly refers to the proportion of the mass of water, oil, or other easily lost substances in the heating process of raw material [30]. In Figure 2, the CLs of all AIR groups were the highest (>11%) while those all of the STE groups were the lowest (<7%), followed by the MIC groups (8~12%). This indicates that the least juice loss came from the steamed surimi gel. CL is mainly influenced by the gel’s network structure. Steaming provides a strong moisture balance between surimi gel and the air surrounding it, resulting relatively low CL. Nevertheless, microwaves cause rapid water vaporization inside the surimi gel. This outward moisture migration tends to increase CL. For air-frying, the circulating hot air takes away moisture from surimi gel continuously, leading to heavy CL. Therefore, steamed surimi gel preserved high amount of moisture, helping improve the gel properties of the product. Practically, steaming could be a suitable cooking method for producing surimi products with elastic properties and juiciness.

Under the same cooking method, HIPE groups showed lower CL than the oil one, especially in STE and MIC samples. Zhang et al. also reported that fat-replaced sausage added with emulsions stabilized by zein/carboxymethyl dextrin had a lower CL compared to sausages with pork back fat under water bath pot (85 °C, 30 min) [31]. As aforementioned, HIPEs can uniformly and stably disperse in the gel network, alleviating severe oil aggregation, which could lower the isolation of MP by oil and provide enough MP cross-linking to form a good gel network to hold juicy substances. Gel-like HIPEs also can be embedded into a gel matrix, thereby stabilizing the structure and preventing moisture movement during heating [32]. These together could distinctively decrease surimi gel CL.

#### 3.1.3. Color Properties

In both the HIPE and oil samples, the AIR groups always exhibited the highest a* or b* value and lowest L* value (Table 3), indicating a yellowish brown and less bright surface. The Maillard reaction is an important approach, causing browning and darkness in foods [33], but it mainly occurs under dry conditions. Steaming provides large amount of moisture on food surfaces, and microwave heating leads to a wet surface due to the continuous occurrence of water from inside the food. Therefore, the possibility for a Maillard reaction to occur and cause darkness and browning under these two cooking styles is quite low. Oppositely, air-frying creates a dry environment with a high temperature, which is suitable for Maillard reactions to occur, promoting food browning. On the other hand, light reflection usually becomes diffuse on rough food surfaces, leading to a decrease in brightness [34]. The AIR groups exhibited more wrinkled surfaces than the other two (Figure S1), which might enhance the decline in brightness.

Using the same cooking method, HIPE groups showed higher L* values compared with their oil counterparts, especially for STE samples. These suggest that surimi added with AKO-HIPEs could increase the brightness of the final gel. Sun et al. also reported a higher brightness in surimi gel-added HIPEs than liquid oil containing soybean oil and Litsea cubeba oil [35]. The AKO-HIPEs dispersed in surimi gel uniformly to increase the light reflection and brightness of the surimi gel.

#### 3.1.4. Water Distribution

**Figure 3:**
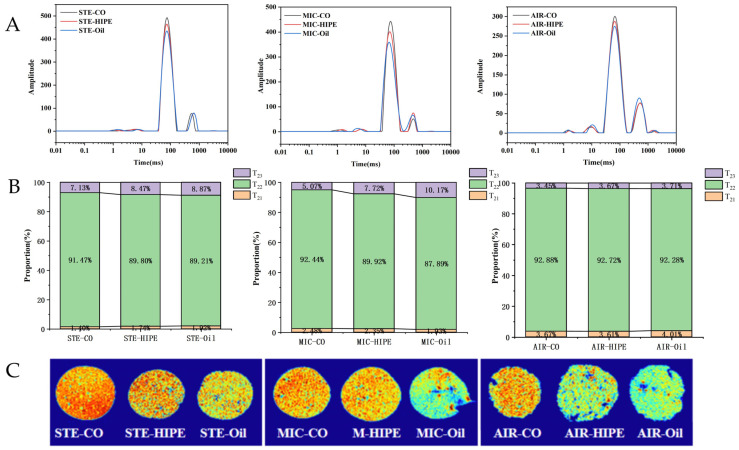
The moisture status (**A**), the T_2_ peak area proportion (**B**), and pseudo-color image (**C**) of surimi gels in different cooking methods. The red spot represents a high hydrogen proton density, and the blue spot represents a low hydrogen proton density (**C**). For abbreviations, see Figure 1.

As shown in Figure 3A, all samples showed three peaks, among which the signal intensity of peak T_22_ was the highest, accounting for more than 85% of the total signal. AIR groups always showed the highest proportion of T_21_ or T_22_ but the lowest T_23_ (Figure 3B). It is likely that the circulation of hot air in air-frying removes a large amount of free moisture, thus increasing the relative proportion of bound water and immobilized water. In Figure 3C, AIR images showed more blue or green areas but less orange area than the STE and MIC groups, indicating lower proton intensity in the AIR group, namely, lower moisture. This could be attributed to the high moisture loss caused by strong air-heating. On the other hand, sharp and fast water loss led by hot air circulation might disrupt the gel’s network structure, decreasing water-holding capability. Nevertheless, STE surimi was heated with sufficient steam; thus, the moisture pressure was balanced inside and outside the gel. This alleviates the damage to the gel network caused by sharp moisture rushing out and preserves a good gel structure with strong water-binding capability. Noticeably, MIC samples showed less pronounced orange area than STE ones. It is known that microwave heats the water inside food intensely. The breaking out of moisture might cause partial gel network collapse, leading to declined water-binding ability.

Compared to the oil groups, the peak T22 of the HIPE groups always exhibited higher amplitude and area proportion regardless of the cooking methods (Figure 3). HIPE groups always exhibited more orange but less blue spots than the oil ones in pseudo-color images, indicating their stronger water-binding capability. HIPEs could be evenly dispersed in surimi to reduce the intervention of lipids on gelation and blockage of the MP cross-linking by oil addition, resulting in enhancements in water stability and proportion in surimi gel [36].

#### 3.1.5. Gel Microstructure

**Figure 4:**
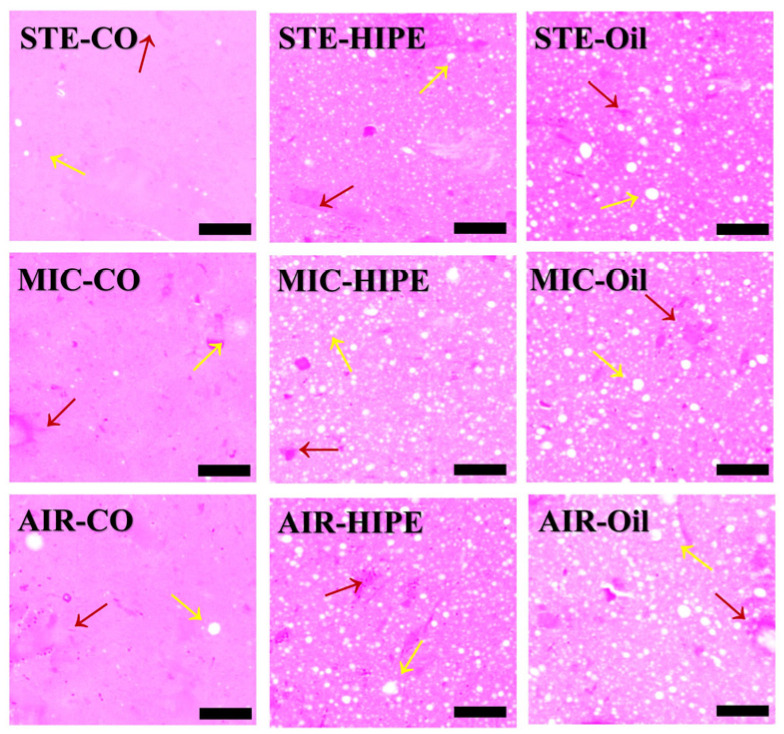
The microstructure of surimi gels in different cooking methods. Scale bar = 100 μm. Yellow arrows indicate oil droplets; red arrows highlight the irregular gel network structure.

Figure 4 shows the microstructure of surimi gel after hematoxylin–eosin staining. The light purple areas are protein gel while the irregular heavy purple areas are gel voids. The white spots are oil droplets. STE samples exhibited even microstructure with few oil droplets. However, MIC samples presented visible gel voids, a slightly rough microstructure, and a large number of emulsion droplets, and AIR samples showed a loose microstructure with low gel homogeneity as well as many oil droplets. As aforementioned, steaming can pass heat uniformly to the interior of surimi gel along with a moisture equilibrium, which reduces the deterioration of gel network caused by moisture diffusion. However, when heated by microwave, the moisture in surimi gel vaporizes rapidly and rushes out the gel to cause sharp damage to the gel network, leading to more inhomogeneous voids [37]. Similarly, an air-fryer can provide intense circulating hot air to lead to a rapid migration of internal moisture to the surface, which would partially destroy the gel network. Moreover, the disruption of the gel network leads to bad accommodation of oil droplets that might aggregate.

All HIPE groups exhibited more homogeneous microstructures with smaller voids and emulsion droplets compared to oil gels. It has also been reported that surimi added with emulsion gels stabilized by tilapia MP had smaller interstitial spaces and fewer oil aggregates, resulting in an improved gel microstructure compared to gels included oil directly [38]. Liquid oil tends to aggregate to form large oil droplets, hindering MP cross-linking. However, emulsion droplets can uniformly and stably disperse in gel network, which could reduce aggregation and alleviate the blocking of protein cross-linking [39]. Additionally, HIPEs, being inherently self-supporting, could serve as fillers into voids to stabilize structure [21].

### 3.2. Lipids Properties of Gels

#### 3.2.1. Fatty Acid

**Figure 5:**
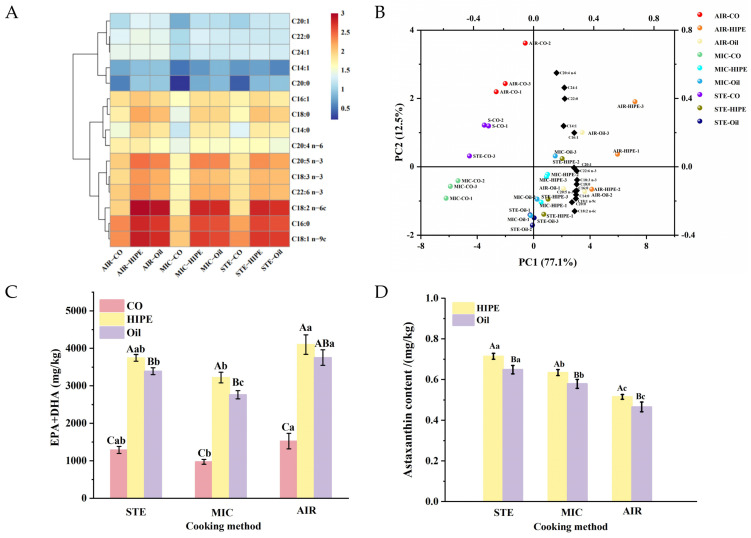
The heat map (**A**) and PCA biplot (**B**) of fatty acids, EPA + DHA (**C**), and astaxanthin (**D**) of surimi gels using different cooking methods. Uppercase letters denote statistical differences between groups of different addition methods in the same cooking method. Lowercase letters denote statistical differences between groups of different cooking methods in the same addition method (*p* < 0.05). For abbreviations, see Figure 1.

Figure 5A and Table 4 show the fatty acid contents of the surimi gels. AKO-HIPEs or AKO-SO addition significantly modified the fatty acid content, especially C18:2 n-6c, C16:0, C20:0, and C20:5 n-3. HIPE groups showed deeper red or orange blocks, suggesting higher content of fatty acids than the corresponding oil groups. Meanwhile, the HIPE and oil groups were separated well along PC1, indicating clear differentiation (Figure 5B). These suggest the protective effect of AKO-HIPEs on FAs. Oil might accumulate in surimi to form large oil droplets, which are more likely to suffer oxidation. However, HIPE droplets are tightly stacked to provide strong oxygen isolation [11]. Therefore, FAs in HIPEs suffer less oxidization, which could alleviate FA loss during cooking. The AIR group is located above the positions of the MIC and STE groups (Figure 5B), indicating the differentiation between them. The FA content in the AIR groups was greater than that of the STE and MIC groups (*p* < 0.05). In fact, the high CL led by heavy loss of moisture in AIR samples might provide a concentrated mass, therefore showing higher contents of FA.

EPA and DHA are important n-3PUFA for human health; thus, EPA + DHA content was calculated (Figure 5C). Compared to oil groups, HIPE groups showed higher EPA + DHA content with the same cooking method. This suggests that HIPEs could inhibit the loss of EPA and DHA during cooking, which may be attributed to the oxygen-isolating impact due to their dense accumulation. Additionally, the presence of the protein film at the HIPE interface can function as a protective barrier, blocking the entrance of oxygen to reduce oxidation [12].

Noticeably, STE-HIPE, MIC-HIPE, and AIR-HIPE had 3744, 3219, and 4099 mg/kg gel of EPA + DHA, respectively, indicating air-fired surimi retained the highest EPA + DHA content. This could be attributed to the high cooking loss under air-frying, which relatively increased EPA + DHA proportion. A daily intake of approximate 500 mg/day DHA + EPA is recommended for adults [40,41]. Based on the results, the consumption of approximately 140, 160, and 128 g STE, MIC, and AIR AKO-HIPE surimi gel can fulfill the daily requirement for EPA + DHA, demonstrating their potential for enhancing human health.

#### 3.2.2. Astaxanthin

Astaxanthin is a natural carotenoid with excellent antioxidative, anti-inflammatory, and immunomodulatory functions. However, astaxanthin is rapidly degraded when exposed to oxygen, light, and heat [42]. In Figure 5D, the astaxanthin contents of HIPE groups were all higher than those of oil groups, indicating the protective effect of HIPEs on astaxanthin during cooking.

The highest astaxanthin content was observed in the STE-HIPE group (0.71 mg/kg) while the lowest was found in the AIR group (0.52 mg/kg). It is possible that the drastic air circulation in air-frying might promote the contact of HIPEs with air, accelerating the oxidation of astaxanthin. On the other hand, microwave might provide casein particles enough energy to escape from the interface, hereby destabilizing HIPEs to allow for more astaxanthin degradation at high temperatures [43]. Nevertheless, steaming creating a circumstance occupied with large amount warm steam, which would effectively prevent the contact of air from the surimi gel, therefore inhibiting astaxanthin loss.

#### 3.2.3. ESR and TBARS

**Figure 6:**
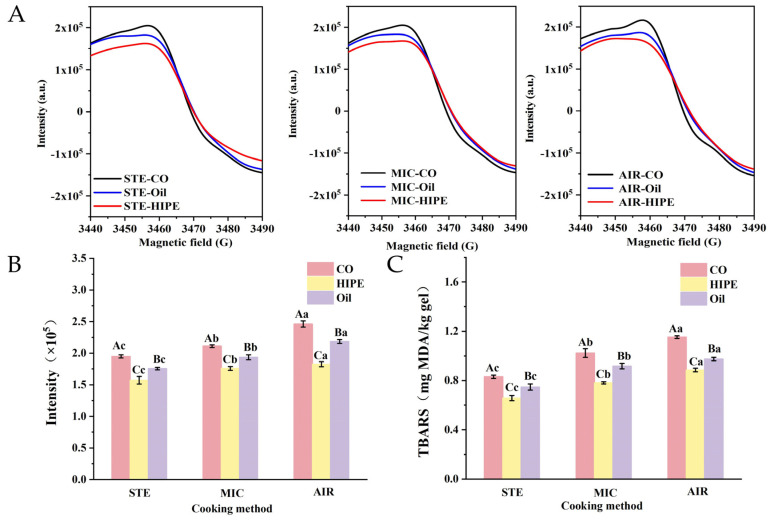
Effect of different cooking methods on the free radical ESR spectrum (**A**), ESR signal intensity (**B**), and TBARS (**C**) of surimi gels. Uppercase letters denote statistical differences between groups of different addition methods with the same cooking method. Lowercase letters denote statistical differences between groups of different cooking methods with the same addition method (*p* < 0.05). For abbreviations, see Figure 1.

As shown in Figure 6, CO always showed higher free radical intensity and TBARS value than HIPE and oil groups with the same cooking methods. Astaxanthin is a powerful antioxidant with strong free radical quenching capability. Thus, it might alleviate the lipid oxidation in surimi gel. Additionally, HIPE group showed lower free radical intensity and TBARS value than oil group, suggesting the alleviated lipid oxidation. The highly accumulated HIPEs could distribute in surimi gel evenly and fill the surimi matrix, reducing the probability of oxygen entering the surimi gel [35].

It was found that the surimi gels of MIC and AIR groups showed higher free radical intensity and TBARS values than STE groups in both HIPE and oil samples. In air-frying, intense air circulation gives gel full contact with air, promoting lipid oxidation [44]. In contrast, steaming uses steam as the heat transfer medium, which reduces air contact to alleviate lipid oxidation. Microwave heating does not involve vigorous air exchange as air-frying does. Therefore, the lipid oxidation caused by microwave heating is less pronounced than air frying. The TBARS values of samples were lower than 2 mg/kg for the samples, denoting they were acceptable to consumers [45]. It has to be pointed that commercial surimi containing approximately 3% cryoprotectants was used in this experiment. The TBARS value may be underestimated due to the interference of sugar.

### 3.3. Electronic Nose

**Figure 7:**
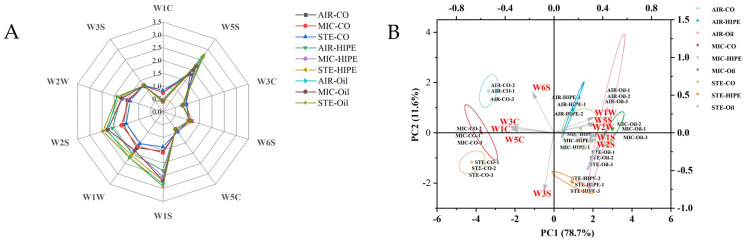
Radar diagram (**A**) and PCA biplot (**B**) by electronic nose of surimi gels with different cooking methods. For abbreviations, see Figure 2.

In Figure 7A, after the addition of AKO-HIPEs and AKO-SO, the response of surimi gels to sensor W1S (methyl groups) and W2S (alcohols, aldehydes, and ketones) increased significantly, leading to a change in the shape of radar fingerprint graph. The response of W5S (nitrogen oxides), W1S, W1W (inorganic sulfides), and W2S was greater in oil groups compared to the corresponding HIPE groups. PCA demonstrates that the oil group was located to the right of HIPE group close to W5S, W1S, W1W, W2S, and W2W (sensitive to organic sulfides) (Figure 7B), indicating that the oil group has a richer flavor than the HIPE group. HIPE droplets are covered by protein, thus could reducing the formation of compounds via lipid oxidation [46].

In Figure 7B, most of the AIR and MIC groups were located in the first and second quadrants, close to aromatic compounds (W3C, W1C, W5C, etc.) while STE groups were located in the third and fourth quadrants, close to the alkane components (W3S). These results indicated that cooking methods significantly changed the aroma profiles of the surimi products. The Maillard reaction, a key factor in production of flavor compounds, is closely related to the cooking method used [47]. When cooked by air-frying, hot air circulation removes moisture from food surface to maintain a dry condition that is favorable for the Maillard reaction to occur. However, when surimi gel is steamed, the surface of gel is too wet to allow for the Maillard reaction. Additionally, a microwave oven also creates a fairly wet gel surface, resulting in a less pronounced Maillard reaction. Thus, air-frying could be an ideal method for cooking surimi products with strong flavor, attractive color, and soft texture.

### 3.4. Sensory Evaluation

**Figure 8:**
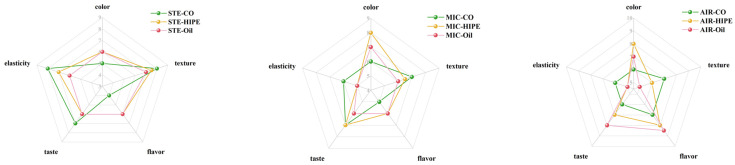
Sensory evaluation of surimi gels using different cooking methods. For abbreviations, see Figure 2.

Figure 8 shows the radar chart of sensory evaluation of surimi products using different cooking methods. The color scores for the HIPE and oil groups were significantly higher than that of CO, which was due to the presence of astaxanthin. The elasticity and texture scores of the HIPE group were higher than the oil group using the same cooking method, which could be attributed to the uniform gel network fortified by AKO-HIPEs during processing (Figure 1 and Figure 4). The flavor score of the group modified by AKO was higher than that of CO, indicating that the flavor of the surimi products was effectively enhanced by AKO due to lipids as well as the flavor compounds generated during cooking. In HIPE groups, the texture scores for STE, MIC, and AIR groups were 7.5, 6.5, and 6, separately. This can be explained by their different gel microstructures (Figure 4). The flavor score of AIR-HIPE was highest while that of STE-HIPE group was lowest, which was in line with the findings of electronic nose (Figure 7A,B). In summary, steaming and air-frying could be good cooking method for surimi.

## 4. Conclusions

AKO-HIPE addition improved the texture properties, water-holding capacity, and microstructure of surimi products. AKO-HIPEs also increased the stability of FAs and astaxanthin in the product. STE enabled good gel characteristics and stabilized n-3 PUFAs and astaxanthin in surimi products while AIR strengthened the color and flavor properties of surimi gel added with AKO-HIPEs. Thus, AKO-HIPEs along with STE or AIR could provide surimi products with fairly good gel properties, flavors, as well as nutrients.

## Figures and Tables

**Table 1:** Experimental design of this study (n = 3).

	CO (No Oil)	Oil	HIPE
Steaming (STE)	STE-CO	STE-Oil	STE-HIPE
Microwave heating (MIC)	MIC-CO	MIC-Oil	MIC-HIPE
Air-frying (AIR)	AIR-CO	AIR-Oil	AIR-HIPE

**Table 2:** Organoleptic attributes and corresponding description and scoring criteria of surimi gels.

Attribute	Operation	Standard for Evaluation	Score
color	Cut the surimi gel into slices (≈10 mm thick) and observe the color of the slices	Uniform color, shiny, and looks appetizing	8–10
		The color is more uniform, shiny, and acceptable	4–7
		Uneven color, no luster, and does not look appetizing	1–3
texture	Cut the surimi gel into slices (≈10 mm thick) and observe the section of the slices	Smooth, dense, and small holes	8–10
		Smooth, dense, and some holes	4–7
		Rough, loose, and many holes	1–3
flavor	Smell the aroma of surimi gels through the nose	Rich aroma	8–10
		The aroma is moderate	4–7
		The aroma is less or abnormal	1–3
taste	Feel the changes of surimi gels during chewing through the tongue	Delicate, silky, easy to chew, no woody feeling	8–10
		Relatively delicate and easy to chew, no woody feeling	4–7
		Rough taste and woody feeling	1–3
elasticity	Press the surimi gel with fingers to feel its elasticity	Good elasticity	8–10
		Moderate elasticity	4–7
		Poor elasticity	1–3

**Table 3:** The color of surimi gel-fortified lipids as HIPEs or oil using different cooking methods.

		L*	a*	b*
STE	CO	80.74 ± 0.44 ^Aa^	−2.18 ± 0.02 ^Ca^	8.16 ± 0.33 ^Cb^
	HIPE	78.83 ± 0.18 ^Ba^	7.18 ± 0.07 ^Bb^	19.13 ± 0.12 ^Bb^
	Oil	78.27 ± 0.24 ^Ca^	7.32 ± 0.03 ^Ab^	19.4 ± 0.06 ^Ab^
MIC	CO	79.95 ± 0.42 ^Aa^	−2.23 ± 0.04 ^Ca^	8.01 ± 0.43 ^Bb^
	HIPE	78.51 ± 0.25 ^Ba^	7.07 ± 0.05 ^Bb^	18.73 ± 0.33 ^Ab^
	Oil	77.81 ± 0.42 ^Ba^	7.21 ± 0.03 ^Ac^	19.51 ± 0.61 ^Ab^
FIR	CO	78.58 ± 0.57 ^Ab^	−2.36 ± 0.06 ^Bb^	10.26 ± 0.57 ^Ba^
	HIPE	72.20 ± 0.61 ^Bb^	9.52 ± 0.43 ^Aa^	26.58 ± 0.25 ^Aa^
	Oil	71.85 ± 1.11 ^Bb^	9.41 ± 0.6 ^Aa^	27.1 ± 0.99 ^Aa^

Uppercase letters denote statistical differences between groups of different addition methods using the same cooking method. Lowercase letters denote statistical differences between groups of different cooking methods using the same addition method (*p* < 0.05).

**Table 4:** Fatty acids composition of surimi gel (mg/100 g) (n = 3).

FA	STE-CO	STE-HIPE	STE-Oil	MIC-CO	MIC-HIPE	MIC-Oil	AIR-CO	AIR-HIPE	AIR-Oil
C14:0	16.0 ± 1.8 ^Bb^	61.3 ± 0.3 ^Ab^	60.5 ± 2.0 ^Ab^	13.3 ± 1.2 ^Cc^	63.5 ± 2.5 ^Ab^	55.1 ± 2.2 ^Bc^	20.0 ± 3.2 ^Ca^	80.5 ± 11.7 ^Aa^	68.3 ± 5.8 ^Ba^
C14:1	2.5 ± 0.5 ^Ba^	4.5 ± 0.5 ^Aa^	7.0 ± 3.3 ^Aab^	1.2 ± 0.2 ^Cb^	5.5 ± 1.4 ^Aa^	3.8 ± 0.3 ^Bb^	3.3 ± 0.6 ^Ba^	6.1 ± 1.7 ^Aa^	8.2 ± 0.9 ^Aa^
C16:0	95.0 ± 16.7 ^Cab^	414.2 ± 4.0 ^Ab^	391.7 ± 8.2 ^Bb^	73.3 ± 10.1 ^Cb^	438.3 ± 23.2 ^Aab^	386.1 ± 20.9 ^Bb^	102.4 ± 23.4 ^Ca^	574.4 ± 125.7 ^Aa^	497.4 ± 42.5 ^Ba^
C16:1	31.6 ± 4.9 ^Ba^	76.4 ± 1.1 ^Aa^	75.2 ± 7.6 ^Aa^	20.0 ± 4.3 ^Ca^	79.4 ± 5.9 ^Aa^	58.1 ± 10.2 ^Bb^	29.3 ± 8.7 ^Ca^	94.4 ± 24.6 ^Aa^	83.8 ± 5.6 ^Ba^
C18:0	25.2 ± 5.6 ^Cb^	93.5 ± 2.5 ^Ac^	86.6 ± 2.1 ^Bc^	17.5 ± 2.8 ^Cb^	109.7 ± 6.2 ^Ab^	94.6 ± 0.9 ^Bb^	35.7 ± 6.1 ^Ca^	143.6 ± 24.6 ^Aa^	123.3 ± 13.1 ^Ba^
C18:1 n-9c	108.7 ± 22.0 ^Ca^	460.7 ± 8.2 ^Ab^	415.1 ± 23.1 ^Bc^	74.9 ± 10.6 ^Cb^	496.2 ± 13.6 ^Aab^	461.0 ± 4.5 ^Bb^	144.9 ± 22.9 ^Ca^	656.6 ± 148.5 ^Aa^	591.5 ± 54.8 ^Ba^
C18:2 n-6c	71.4 ± 4.3 ^Cb^	605.7 ± 12.1 ^Ab^	569.6 ± 4.9 ^Bb^	50.6 ± 8.4 ^Cc^	633.8 ± 18.7 ^Ab^	539.7 ± 5.2 ^Bc^	93.0 ± 15.2 ^Ba^	875.5 ± 88.6 ^Aa^	788.4 ± 68.0 ^Aa^
C18:3 n-3	36.9 ± 4.9 ^Ca^	124.7 ± 0.9 ^Aa^	114.8 ± 2.2 ^Bb^	13.5 ± 3.0 ^Bb^	131.5 ± 7.4 ^Aa^	120.8 ± 7.4 ^Ab^	42.2 ± 8.5 ^Ca^	159.9 ± 44.4 ^Aa^	145.1 ± 9.9 ^Ba^
C20:0	2.4 ± 0.5 ^Ba^	5.9 ± 0.3 ^Ab^	7.1 ± 2.1 ^Ab^	1.6 ± 0.5 ^Ba^	7.4 ± 0.2 ^Aa^	8.1 ± 1.4 ^Aa^	2.5 ± 0.7 ^Ba^	9.8 ± 3.5 ^Aa^	9.2 ± 1.3 ^Aa^
C20:1	5.5 ± 1.4 ^Ca^	14.5 ± 0.3 ^Ab^	13.2 ± 0.2 ^Bb^	3.1 ± 0.6 ^Bb^	16.4 ± 2.3 ^Aab^	17.2 ± 1.1 ^Aa^	6.2 ± 1.4 ^Ba^	20.1 ± 3.9 ^Aa^	19.5 ± 3.4 ^Aa^
C20:4 n-6	30.8 ± 3.6 ^Ca^	41.7 ± 1.0 ^Aa^	37.1 ± 0.7 ^Ba^	27.3 ± 1.8 ^Ca^	43.0 ± 6.1 ^Aa^	30.2 ± 0.8 ^Bb^	30.4 ± 5.4 ^Ca^	48.5 ± 12.8 ^Aa^	38.8 ± 2.0 ^Ba^
C22:0	10.0 ± 1.3 ^Ca^	20.2 ± 0.4 ^Aa^	18.0 ± 0.6 ^Ba^	11.5 ± 0.8 ^Ba^	17.2 ± 2.4 ^Aa^	17.6 ± 0.1 ^Aa^	12.7± 2.0 ^Ba^	22.8 ± 6.5 ^Aa^	23.0 ± 7.8 ^Aa^
C20:5 n-3	69.1 ± 4.4 ^Bb^	227.7 ± 4.6 ^Ab^	201.1 ± 6.2 ^Ab^	49.4 ± 3.3 ^Cc^	205.6 ± 6.4 ^Ac^	157.6 ± 3.4 ^Bc^	85.7 ± 10.6 ^Ca^	253.7 ± 12.3 ^Aa^	230.9 ± 12.6 ^Ba^
C24:1	12.2 ± 1.6 ^Aa^	10.6 ± 2.7 ^Aa^	9.5 ± 4.4 ^Aa^	9.0 ± 0.8 ^Ab^	10.3 ± 1.3 ^Aa^	9.7 ± 1.7 ^Aa^	14.7 ± 2.4 ^Aa^	11.0 ± 6.9 ^Aa^	9.3 ± 3.2 ^Aa^
C22:6 n-3	60.1 ± 5.0 ^Ca^	146.7 ± 4.3 ^Aa^	138.0 ± 3.0 ^Bb^	48.0 ± 3.0 ^Cb^	136.4 ± 7.8 ^Aa^	118.7 ± 7.4 ^Bc^	66.9 ± 10.1 ^Ca^	156.2 ± 13.7 ^Aa^	144.4 ± 8.1 ^Ba^

Uppercase letters denote statistical differences between groups of different addition methods with the same cooking method. Lowercase letters denote statistical differences between groups of different cooking methods with the same addition method (*p* < 0.05).

## Data Availability

The original contributions presented in this study are included in the article/Supplementary Materials. Further inquiries can be directed to the corresponding author.

## Supplementary Materials

The following supporting information can be downloaded at: https://www.mdpi.com/article/10.3390/foods13244070/s1, Figure S1: Effect of different cooking methods on appearance of surimi gels fortified lipids using HIPEs- or oil-added methods.
