# Supplementary material for: The Effects of Cooking Methods on Gel Properties, Lipid Quality, and Flavor of Surimi Gels Fortified with Antarctic Krill (Euphausia superba) Oil as High Internal Phase Emulsions
Source: Foods. 2024 Dec 17;13(24):4070. doi: 10.3390/foods13244070 (PMC11675233; doi:10.3390/foods13244070)

**Supplementary materials**

Figure S1: Effect of different cooking methods on appearance of surimi gels fortified lipids by HIPEs- or Oil-added way.


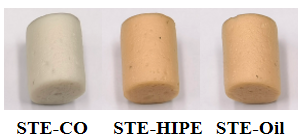

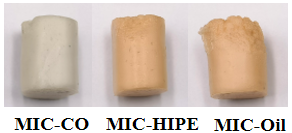

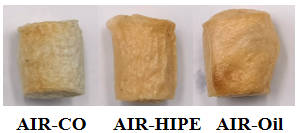

Supplement: Supplementary file 1 [file foods-13-04070-s001.zip › Supplementary materials.docx]
